# Reduced Expression of Galectin-9 Contributes to a Poor Outcome in Colon Cancer by Inhibiting NK Cell Chemotaxis Partially through the Rho/ROCK1 Signaling Pathway

**DOI:** 10.1371/journal.pone.0152599

**Published:** 2016-03-30

**Authors:** Yang Wang, Jintang Sun, Chao Ma, Wenjuan Gao, Bingfeng Song, Hao Xue, Weiliang Chen, Xi Chen, Yun Zhang, Qianqian Shao, Qingjie Wang, Lei Zhao, Jia Liu, Xiuwen Wang, Huayang Wang, Yun Zhang, Meixiang Yang, Xun Qu

**Affiliations:** 1 Institute of Basic Medical Sciences, Qilu Hospital, Shandong University, Jinan, Shandong, People's Republic of China; 2 Department of Chemotherapy, Qilu Hospital, Shandong University, Jinan, Shandong, People's Republic of China; 3 The Key Laboratory of Cardiovascular Remodeling and Function Research, Chinese Ministry of Education and Chinese Ministry of Health, Qilu Hospital, Shandong University, Jinan, Shandong, People's Republic of China; INRS, CANADA

## Abstract

Galectin-9 is a widely expressed protein that is involved in immune regulation and tumorpathogenesis and serves as a marker of a poor prognosis in various types of cancers. However, the clinical impact and the precise mechanism by which this protein contributes to colon tumor progression are unclear. In the present study, we detected the expression of galectin-9 and CD56 cells using immunohistochemistry. Spearman's rank correlation was used to clarify the association between galectin-9 expression and natural killer (NK) cell infiltration. The influence of galectin-9 on NK-92 cell migration was evaluated in vitro using transwell chemotaxis assays. The role of rh-galectin-9 in F-actin polarization in NK-92 cells was investigated using laser scanning confocal microscopy. We showed that galectin-9 was expressed in 101 (78.91%) colon tumor tissues and that was expressed at lower levels in these tissues than in para-tumor tissues. Low levels of galectin-9 expression were positively correlated with a poor histological grade and lymph node metastasis (P<0.05). A Kaplan-Meier method and Cox proportional hazards regression analysis showed that overall survival was longer in patients with high galectin-9 expression in an 8-year follow-up (P<0.05). Spearman's rank correlation indicated that there was a linear correlation between galectin-9 expression and CD56^+^ NK cell infiltration (R^2^ = 0.658; P<0.0001). Galectin-9 stimulated migration in human NK-92 cells by affecting F-actin polarization through the Rho/ROCK1 signaling pathway. These results suggest that galectin-9 expression potentially represents a novel mechanism for tumors to escape immune surveillance in colon tumors.

## Introduction

Each year, approximately 1.2 million patients develop colorectal cancer (CRC)and 600,000 individuals die from this disease around the world [1]. Despite the fact that there have been positive improvements in surgical and pharmaceutical strategies, CRC remains far from therapeutic control[2]. The present dearth of knowledge regarding the immunological and molecular underlying causes of CRC is a major obstacle to improving treatments for this disease.Hence identifying new biomarkers is necessary to the future development of targeted CRC therapies.

The development of cancer is a multi-step process that is governed not only by numerous cell intrinsic factors but also by extrinsic factors in the tumor microenvironment[3, 4]. As important components of the tumor microenvironment, certain types of leukocytes influence tumor progression and prognosis[5–7]. Natural killer (NK) cells are one of the major cell types in the innate immune system. In CRC, extensive intratumoral infiltration by NK cells is associated with a better prognosis, depending on their cytotoxic effects on cancer cells[8, 9]. However, a recent study found that NK cells are generally scarcer in the CRC microenvironment than in adjacent normal mucosa despite the presence of relatively high levels of NK cell-responding chemokines in tumor tissues [10]. This contradiction suggested that chemokines alone might not be sufficient to recruit NK cells to the tumor.

Galectins are soluble members of the lectin superfamily that are characterized by the presence of a carbohydrate recognition domain and β-galactoside binding affinity. A total of 15 mammalian galectins have been so far identified[11]. Among these galectins, galectin-9 exhibits immunoregulatory effects through which it interferes with the function and biological behaviors of various types of immune cells, including T cells, dendritic cells and NK cells[12, 13]. In tumor-bearing mice, galectin-9 increased the number of NK cells in the peritoneal exudate[14], indicating that it plays a potential regulatory role that involves NK cells during tumor progression. In particular, lower levels of galectin-9 have been observed in most types of cancer cells, including oral squamous cell carcinoma[15], melanoma[16], breast cancer [17] and gastric cancer[18], than in their normal counterparts. Given the close association between galectin-9 expression and NK cell numbers, it is reasonable to speculate that a reduced level of galectin-9 in a tumor contributes to the poor infiltration of NK cells into the tumor microenvironment. However, because the presence and significance of galectin-9 expression has not yet been demonstrated in colon cancer tissues, it remains unclear whether this association occurs in colon cancer and what regulatory mechanisms are involved, if any.

In the present study, we found that galectin-9 expression was reduced in colon tumor tissues, which is associated with poor prognosis in these patients. We also provide evidence using *in vitro* studies that galectin-9 enhances NK cell migration by exerting effects on F-actin polarization via the Rho/ROCK1 signaling pathway. These results represent a potentially novel mechanism through which tumors might escape from immune surveillance.

## Materials and Methods

### Patients and tissues

Our study included data that was obtained from 128 patients with histologically confirmed colon cancer who underwent surgery at the Qilu Hospital of Shandong University from January 2004 to December 2011 (Jinan, Shandong, China),This including one group of 38 patients in which we compared para-tumor with tumor tissue and another group of 90 patients were included in the survival analysis. The collection and use of tissue samples complied with the relevant guidelines and institutional practices of the Ethics Committee of Qilu Hospital of Shandong University, and written approval was obtained in each case before tissue sample collection. The ethics committee of Qilu Hospital of Shandong University approved this study (KYLL-2013-069). The key clinicopathological data are shown in Table 1 and the Supplementary Materials and methods (S1 File).

**Table 1:** Demographic parameters of patients who participated in the present study.

		No. of Patients	N%
Assessable			
	Entered	128	100%
	To compare para-tumor and tumor tissue	38	29.69%
	For the survival analysis	90	70.31%
Gender			
	Male	65	50.78%
	Female	63	49.22%
Age (years)			
	Median (range)	66.5 (24~90)	
ECOG PS			
	0	70	54.69%
	1	16	12.50%
	2	42	32.81%
Tumor differentiation			
	Well	20	15.63%
	Moderate	63	49.22%
	Poor	45	35.15%
Stage of disease at diagnosis			
	I	11	8.59%
	II	66	51.56%
	III	45	35.16%
	IV	6	4.69%

### Immunohistochemistry and Evaluation

Sections (4 μm) of formalin-fixed paraffin-embedded colon tumor tissue samples were dewaxed, rehydrated, and incubated with the following primary antibodies: rabbit polyclonal anti-human galectin-9 (sc-366141, 1:200; Santa Cruz Biotechnology, Santa Cruz, CA, USA) and mouse monoclonal anti-human CD56 (3576, 1:50; Cell Signaling Technology, Beverly, MA,USA). The sections were subsequently stained with the corresponding secondary antibodies. Two independent pathologists who were blinded to the clinical data, evaluated the immunohistochemical results. The method used to evaluate the tissues is described in the Supplementary Materials and methods (S1 File).

### Cells and treatments

Cells were incubated according to the standard procedures described in the ATCC culture guide. The human colon tumor cell lines SW480, SW620 and HT29 were grown in Dulbecco's modified Eagle’s medium (DMEM) (Invitrogen, USA) supplemented with 5% fetal bovine serum (FCS) (Bio International, USA). The human NK92 cell line was a gift from The Second Military Medical University (Shanghai, China) [19] and cultivated with Alpha Minimum Essential medium (Gibco, USA) containing 2 mM L-glutamine and 1.5 g/L sodium bicarbonate but without ribonucleosides and deoxyribonucleosides, and various components were added to obtain complete growth medium: 0.2 mM inositol (17508, Sigma, USA); 0.1 mM 2-mercaptoethanol (ES-007E, Merck Millipore, Germany); 0.02 mM folic acid (F8758, Sigma, USA); 100–200 U/mL recombinant IL-2; and adjustment to a final concentration of 12.5% horse serum (16050, Gibco, USA) and 12.5% fetal bovine serum. All cells were incubated at 5% CO_2_ according to the standard procedures in the ATCC culture guide.

### Isolation of human NK cells

Peripheral blood mononuclear cells (PBMCs) were obtained after centrifugation with Ficoll-Paque Plus (Amersham Biosciences, Sweden). NK cells were isolated using NK Cell Isolation Kits (MiltenyiBiotec, Germany) according the manufacturer’s instructions. The purity of CD3^-^CD56^+^ cells was consistently over 95%, measured through flow cytometric analysis.

### RNA interference

We transiently transfected of siRNA against human galectin-9 (siRNA #378 and siRNA #690, GenePharma, Shanghai, China) or scramble siRNA into HT-29 cells using Lipofectamine 2000 (11668–019, Invitrogen, Carlsbad, CA, USA) according to the manufacturer’s instructions. The knockdown efficacy of the siRNAs targeting galectin-9 was examined using RT-PCR, western blot analysis and ELISA at 36 h post-transfection. The following siRNA sequences targeting human galectin-9 were used:

siRNA378: sense, 5’-GGAAGACACACAUGCCUUUTT-3’; antisense, 5’-AAAGGCAUGUGUGUCUUCCTT-3’; and siRNA 690: sense, 5’-CCAUUACCCAGACAGUCAUTT-3’; antisense, 5’-AUGACUGUCUGGGUAAUGGTT-3’.

### Migration assay

We used transwell chambers with a 5-mm pore membrane (Costar, Corning, NY, USA). NK-92 cells or primary NK cells were plated in the top chambers. Colon tumor cell culture supernatant or varying concentrations of rh-galectin-9 (2045-GA-050, R&D, Minneapolis, MN, USA) were added to the bottom chamber. NK-92 cells or primary NK cells were permitted to migrate into the bottom chamber for 4 h at 37°C, They were then harvested and counted using cell-count boards. Because NK cells have a relatively high rate of spontaneous migration, we used the following the migration index (MI) to evaluate the results: migration index = the number of cells migrated/the number of randomly migrating cells (no chemokine)[20]. Y-27632 dihydrochloride (20 μM) (ab120129, Abcam, Cambridge, Massachusetts,UK) and C3 transferase (2.0 μg/mL) (CT04-A, Cytoskeleton, Denvor, CO, USA) were added to test the role of Rho/ROCK signaling in NK migration. IL-12 (7.5 ng/mL) was used as a positive control for NK cell chemoattraction (200-12P80, Peprotech, Chicago, USA)[21].

### Western blot analysis

Cell lysates were resuspended in a small volume of lysis buffer as previously described and then subjected to western blot analysis[22]. The details of the western blotprocedure are described in the Supplementary Materials and Methods (S1 File).

### Total RNA isolation, cDNA synthesis and quantitative real-time PCR

Total RNA was extracted from cultured/transfected cells using TRIzol reagent (15596026, Invitrogen, USA) and then stored at -20°C. Using aReverTra Ace qPCR RT Kit (FSQ-101, Toyobo, Japan), we reverse transcribed 1 μg of total RNA into DNA in a one-step reaction.This was followed by PCR to amplify the transcripts of the corresponding target molecule. The RT reactions and PCR steps were performed according to the manufacturer’s instructions. Table 2 lists the primer sequences that were obtained from Primer Bank. The target gene expression levels were normalized to GAPDH levels that were run during the same reaction.

**Table 2:** Primer sequences.

mRNA	Forward Primer	Reverse Primer
GAPDH	CCGATGCCTTTCATCACCACC	CACCTTGAGGCAGTGAGCTTC
RhoG	ACTAACGCTTTCCCCAAAGAG	GTGTACGGAGGCGGTCATAC
RhoQ	CCACCGTCTTCGACCACTAC	AGGCTGGATTTACCACCGAGA
RhoH	ATGCTGAGTTCCATCAAGTGC	TCTGCCTGCTGGTAGGACA
RhoT1	AAGGTAACAAGTCGATGGATTCC	TCAGGTTTTTCGCTGAACACT
RhoA	GGAAAGCAGGTAGAGTTGGCT	GGCTGTCGATGGAAAAACACAT
RhoB	ATCCCCGAGAAGTGGGTCC	CGAGGTAGTCGTAGGCTTGGA
RhoC	GGAGGTCTACGTCCCTACTGT	CGCAGTCGATCATAGTCTTCC
RhoF	CCCCATCGGTGTTCGAGAAG	GGCCGTGTCGTAGAGGTTC
Galectin-9	GGTGGTCTCCTCTGACTTCAACAG	GTTGCTGTAGCCAAATTCGTTGT

### ELISA

The supernatants of HT29 tumor cells were collected at 36h post-transfection. Each supernatant was centrifuged and stored at -80°C.Ahuman galectin-9 ELISA kit(Cusabio, WuHan, HuBei Province, China) was used according to the manufacturer’s instructions to measure galectin-9 production in each supernatant.

### Confocal analysis for F-actin in NK cells

NK-92 cells treated with or without rh-galectin-9 for 4 h were added to anti-off slides, fixed in 4% paraformaldehyde and permeabilized using 0.2% Triton X-100 (X100, Sigma-Aldrich, USA). The cells were then incubated with rhodamine-labeled phalloidin (PHDR1, Cytoskeleton, USA) and DAPI (C1002, Beyotime Biotechnology, Shanghai, China) according to the manufacturer’s instructions. Image capturing and processing were performed using a laser scanning confocal microscope (LSM710, ZEISS, Germany).

### Statistical analyses

Survival was measured as the number of months from resection to the last review. The Kaplan-Meier method was used to calculate survival curves, and the log rank test was used for the statistical analyses. The statistical analyses were conducted using Pearson’s χ2 tests, Fisher’s exact tests, t tests and Spearman's rank correlation. A Cox proportional hazards model was used for the univariate and multivariate analyses to evaluate the prognostic value of clinicopathological factors. All tests were two-sided, and P≤0.05 was considered to indicate statistical significance. All statistical analyses were performed using SPSS 17.0 software (IBM Corporation, Armonk, NY, USA).

## Results

### Expression of galectin-9 in CRC tumor tissues

First, we explored galectin-9 expression in colon cancer by analyzing 128 biopsies from colon tumors spanning stages I-IV according to TNM. Among these samples, 101 (78.91%)tested positive for galectin-9 expression, and immunization scores of 1, 2, 3, and 4 were observed in 31.25% (40 out of 128), 18.75% (24 out of 128), 19.53% (25 out of 128) and 9.38% (12 out of 128) of the cases, respectively.

**Fig 1:**
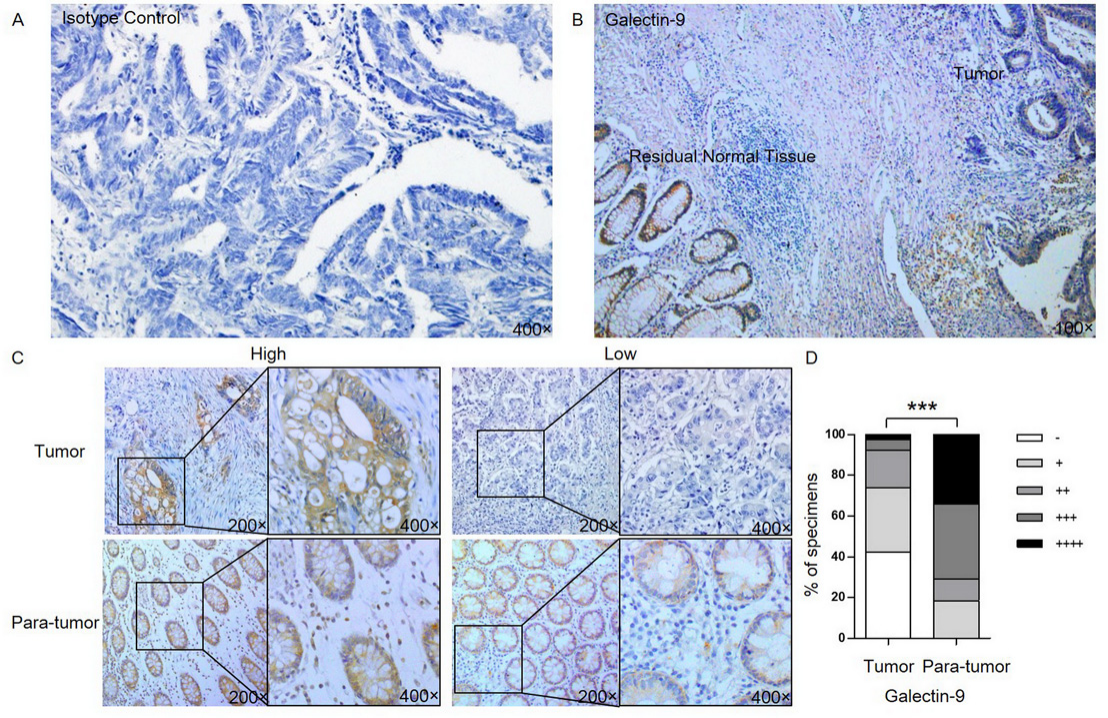
Galectin-9 expression in human CRC. Galectin-9 expression in colon tumor and para-tumor tissue samples was detected using immunohistochemistry using an isotype control (A) or an anti-galectin-9 antibody (B). (C), Representative galectin-9^high^ (left) and galectin-9^low^ (right) expression in tumor and para-tumor tissues. (D), Graphical illustration of the statistical distribution of galectin-9 expression levels in 38 colon cancer tissues and their corresponding normal mucosal samples. The expression intensity was represented as a histological score (∑pi), where p is the percentage of galectin-9 positive cancer cells (scored as 1, 1–10%; 2, 11–50%; 3, 51–80%; or 4, 81–100%) and i represents the staining intensity (scored as 0, no staining; 1, weak staining; 2, moderate staining; or 3, strong staining). Strong (++++), moderate (+++), mild (++), weak (+) and negative (-) staining were 10–12, 7–9, 4–6, 1–3 and 0, respectively.

Our evaluation of tumor and para-tumor tissue samples that were obtained from 38 patients revealed that galectin-9 was expressed primarily in the cytoplasm on both tumor and normal glandular cells but was not expressed in the nucleus or on cells urfaces(Fig 1A–1C). However, the level of galectin-9 expression between the two tissue types was significantly different. In the normal colonic mucosal samples, galectin-9 was expressed in the cytoplasm of normal colon glandular epithelia in all cases. Among the positive cases, 7 had a score of 1 (18%), 4 had a score of 2 (11%), 14 had a score of 3 (37%), and 13 had a score of 4 (34%) (Fig 1D). However, in the corresponding tumor tissue samples, only 58% (22 out of 38 cases) were positive for galectin-9 expression. Moreover, the median immunohistochemistry score for galectin-9 was lower in the colon carcinoma samples than in in the normal mucosal samples (P<0.001, Fig 1D).

### Galectin-9 expression and clinical outcomes in colon cancer patients

We explored the prognostic value of galectin-9 in colon cancer. A total of 90 colon cancer cases and their associate clinical and survival information were analyzed to determine the role of galectin-9 expression. Based on the median of the observed levels of galectin-9, the colon tumor patients were divided into low (0–1, n = 39) and high (2–4, n = 51) expressing groups. Significant differences were found in galectin-9 expression was according to TNM stage, lymph node metastasis, and histological grade but not gender, age, ordistant metastasis (Table 3).

**Table 3:** Association between galectin-9 expression and clinicopathological features in colon cancer.

	Galectin-9 expression			
	n	High (51)	Low (39)	P
Gender				
Male	47	29	18	0.314
Female	43	22	21	
Age	Miss 2			
<70	42	25	17	0.488
≥70	46	24	22	
TNM	Miss 2			
I-II	54	37	17	**0.005**
III-IV	34	13	21	
Lymph node metastasis				
+	34	13	21	**0.006**
-	56	38	18	
Distant metastasis	Miss 1			
+	2	0	2	0.189
-	87	50	37	
Histological grade				**0.022**
I	8	4	4	
II	47	33	14	
III	35	14	21	

**Note:** Pearson’s χ2 and Fisher’s exact tests were used to compare gender, age, TNM stage, lymph node metastasis, distant metastasis and histological grade between patients with and without high galectin-9 expression at tumor sites. Significant correlations (P<0.05) are indicated in bold.

**Fig 2:**
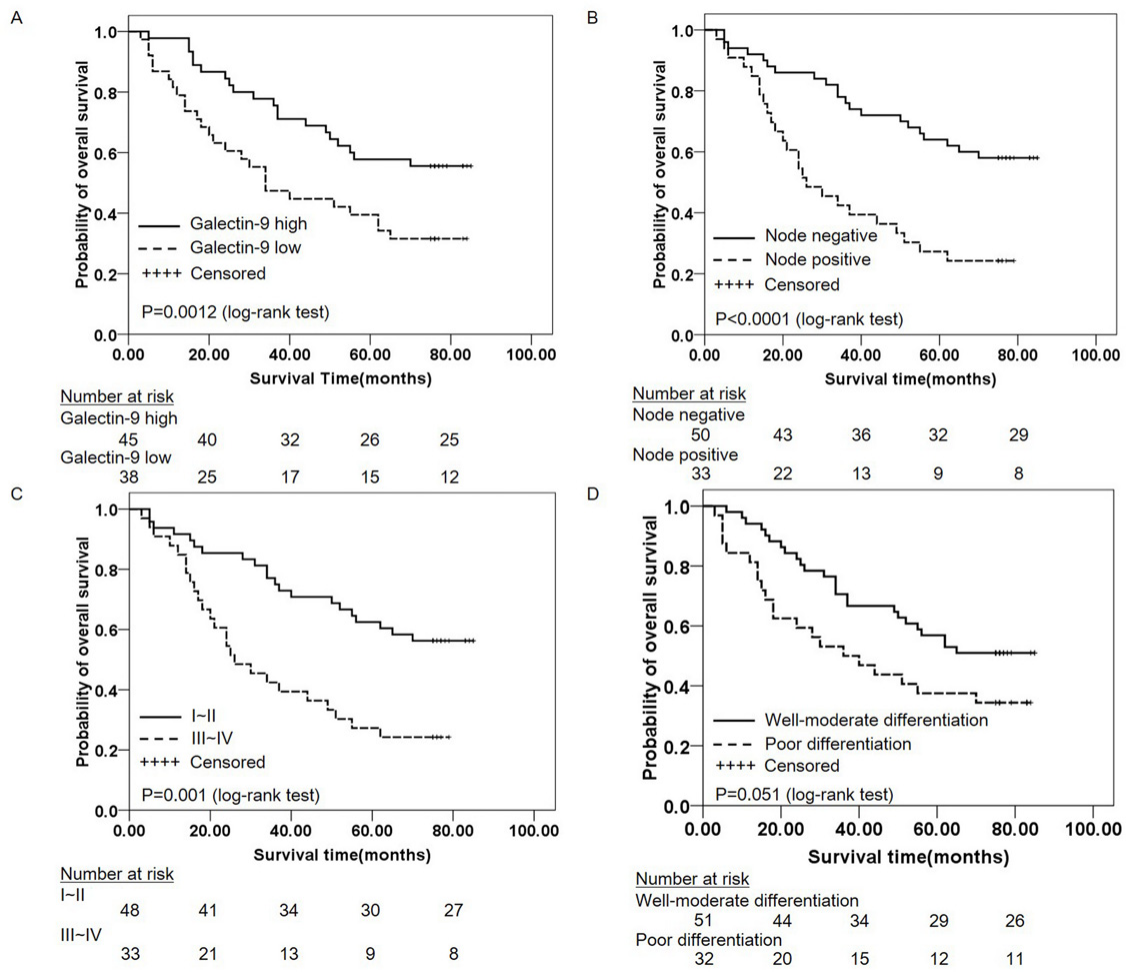
Overall survival curves generated by Kaplan-Meier analysis. Kaplan-Meier curves are shown to describe the relationship between galectin-9 expression and overall survival (A), lymph node metastasis and overall survival (B), TNM stage and overall survival (C), and histological differentiation and overall survival (D). Test sample (n = 90). Galectin-9^high^ expression, no lymph node metastasis and TNM stage (I/II) were found to predict longer survival times (P<0.05, log-rank test).

Univariate analysis of the clinicopathological factors indicated that early TNM stage (I/II) and no lymph node metastasis (N-) were significantly associated with good outcomes (P = 0.001and P = 0.001, respectively). In particular, high galectin-9 expression was significantly associated with good survival (hazard ratio (HR): 0.483; 95% confidence interval (CI): 0.269–0.867; P = 0.015) (Table 4). The median survival times in patients with galectin-9^high^ and galectin-9^low^ were 87 and 34 months, respectively. The survival curves are shown in Fig 2A (log-rank test, P = 0.012). The results of the Kaplan-Meier analyses were consistent with the univariate analysis (Fig 2B and 2C). In addition, overall survival was better in patients with well to moderate differentiation was better than in poorly differentiated cases, but this association did not reach significance (Fig 2D, P = 0.051).

**Table 4:** Prognostic predictors for OS in the univariate and multivariate analyses.

Variables	Univariate analysis			Multivariate analysis		
	HR	95%CI	P	HR	95%CI	P
Age (years) (≥70 vs.<70)	0.591	0.322–1.084	0.089			
Sex (female vs. male)	0.758	0.423–1.358	0.352			
TNM (III-IV vs. I-II)	2.635	1.466–4.736	0.001*	2.371	1.306–4.304	0.005*
Histological grade (III vs. I-II)	1.764	0.987–3.155	0.056			
Galectin-9 (high vs. low)	0.483	0.269–0.867	0.015*	0.549	0.303–0.995	0.048*
Ulcerative type (Yes vs. No)	0.606	0.330–1.113	0.106			

Abbreviation: OS: overall survival, HR: hazard ratio, CI: confidence interval

*Significance value P<0.05.

Moreover, the independent parameters that were found to be significant in the univariate analysis, including TNM stage and galectin-9 expression, were then used in another multivariate analysis. The results suggested that galectin-9 expression in colon tumor tissues (HR: 0.549, 95%CI: 0.303–0.995, P = 0.048) and TMN stage (HR: 2.371, 95%CI: 1.306–4.304, P = 0.005) were independent prognostic markers, demonstrating that galectin-9 expression in colon tumor tissues is a positive prognostic factor for overall survival (Table 4).

### Relationship between the density of CD56^+^ NK cells and galectin-9 expression in CRC tumor cells

**Fig 3:**
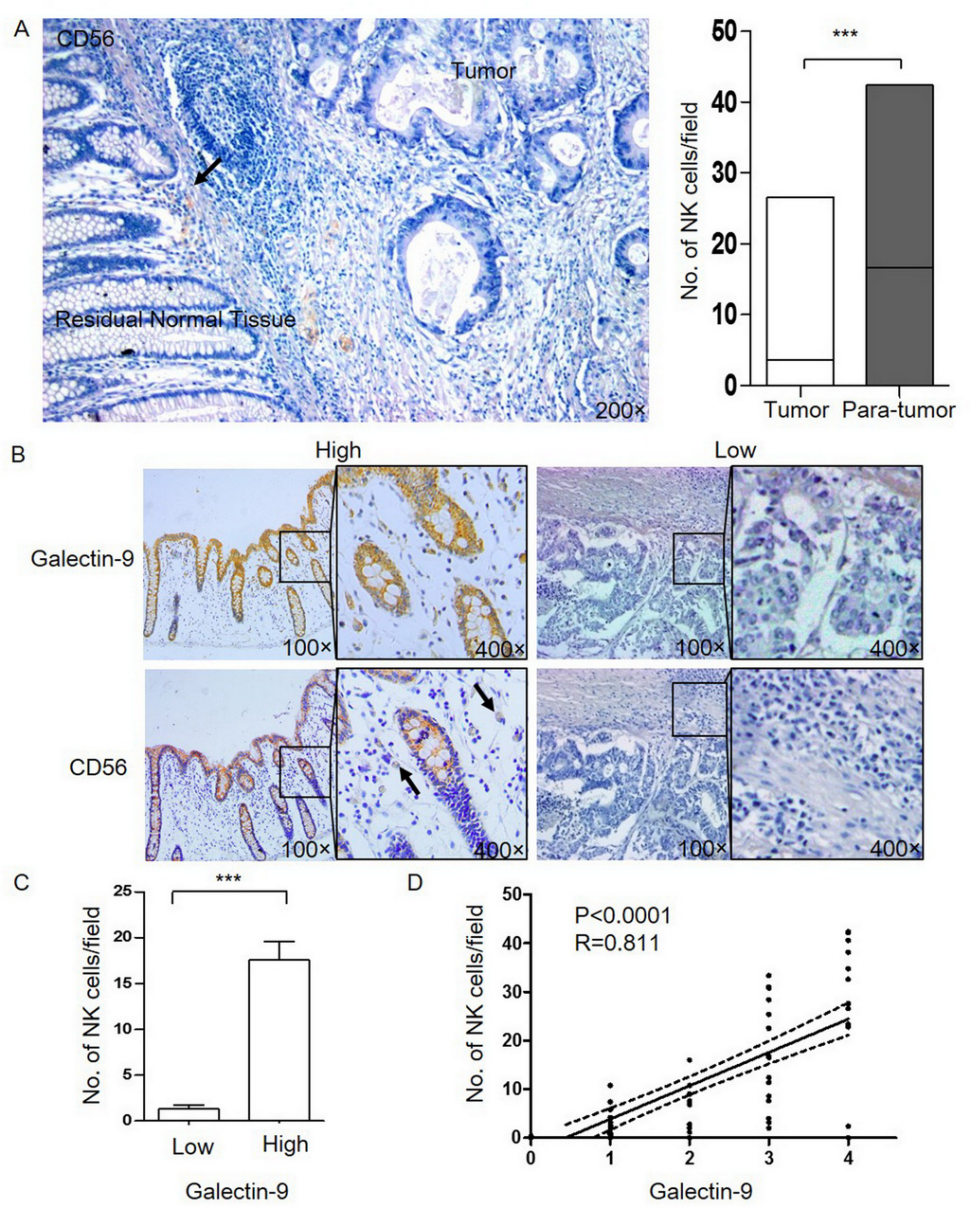
The distribution of CD56^+^NK cells in human CRC tumors is associated with galectin-9 levels. (A), CD56 expression was detected in colon tumor and para-tumor tissue samples (38 cases) using immunohistochemistry with an anti-CD56 antibody or an isotype control. The arrow indicates NK cells; Panels show the numbers of CD56^+^ NK cells that were observed in every microscopic field in the tumor and para-tumor tissues. (B), Representative images from tumors expressing high or low amounts of galectin-9 (top) and CD56 (below) at the same site. (C), Panels showing the numbers of CD56^+^ NK cells that were observed in every microscopic field in galectin-9^high^ or galectin-9^low^ tissue samples. (D), The correlation between galectin-9 and CD56 expression in the tissue samples.

We next investigated the infiltration of CD56^+^ NK cells in colon cancer tissues and its association with galectin-9 expression. CD56^+^ cells were detected within colon cancer cell nests, colon glands and the stroma(Fig 3A). The median number of CD56^+^ cells was 0.3 (range: 0.0~26.6) per single high-power field (HPF) in colon tumor tissue and 16.25 (range: 0.0–42.4) in para-tumor tissues.

Then we analysis the clinical significance of NK cells in 38 colon cancer patients. Based on the median of the NK cells infiltration, the colon tumor patients were divided into negative and positive groups. Significant differences were found in NK cells infiltration according to TNM stage (Table 5).

**Table 5:** Association between NK infiltration and clinic-pathological features in 38 colon cancer.

		CD56 expression		
	n	positive(21)	negative(17)	P
Gender				
Male	18	8	10	0.3275
Female	20	13	7	
Age				
<59	18	10	8	1.000
≥59	20	11	9	
TNM				
I-II	21	16	5	**0.0079**
III-IV	17	5	12	
Lymph node metastasis				
+	16	6	10	0.0990
-	22	15	7	
Histological grade	Miss 1			
Low	9	5	4	0.9905
Moderate	16	9	7	
High	12	7	5	

Note: Fisher’s exact tests were used to compare gender, age, TNM stage, lymph node metastasis and histological grade between patients with and without high NK cells infiltration at tumor sites. Significant correlations (P<0.05) are indicated in bold.

The amount of CD56^+^ cell infiltration was significantly higher in para-tumor tissues than in tumor tissues (P<0.001, Fig 3A). Moreover, the percentage of intratumoral infiltration by CD56^+^ cells was significantly higher in galectin-9^high^ tissues than in galectin-9^low^ tissues (as the numbers of CD56^+^ cells in cancer nests and para-tumoral tissues, median 7.6 vs. 0; range 0–42.4 vs. 0–0.8; P<0.0001) (Fig 3B and 3C). Galectin-9 expression was significantly correlated with CD56^+^ NK cell infiltration in colon cancer and para-tumor samples (P<0.0001, R^2^ = 0.658, Fig 3D)

### Galectin-9 increased chemotaxis in human NK cells

**Fig 4:**
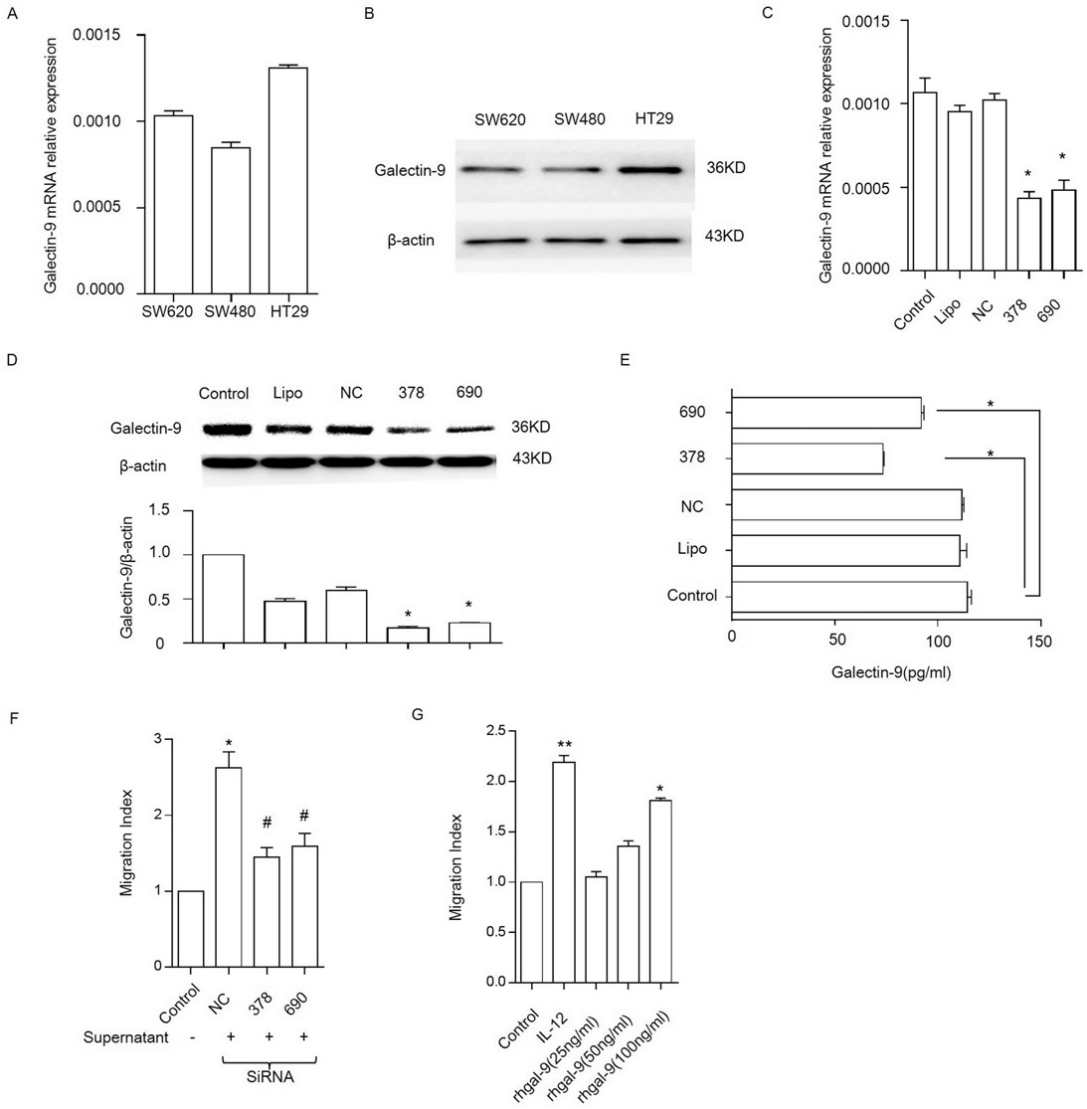
Galectin-9 enhances NK-92 cell chemotaxis. Galectin-9 expression was examined in colon tumor cell lines (HT29, SW480, and SW620) using qRT-PCR (A) and western blot analysis (B). HT29 cells were divided into 5 groups: a control group (cultured with only medium), a lipo group (treated with Lipofectamine 2000), an NC group (transfected with the negative controlsiRNA), a galectin-9-siRNA (#378) group and a galectin-9-siRNA (#690) group. The knockdown efficacies of siRNAs targeting galectin-9 were examined using qRT-PCR (C) and western blot analysis (D). * P<0.05 vs. control. (E), Galectin-9 secretion levels were measured in different groups using galectin-9 ELISA kits. All results are shown as the mean and SEM of quadruplicate experiments.* P<0.05 vs. control.(F), Chemotaxis of NK-92 cells in response to HT29 supernatants treated with galectin-9 siRNA; * P<0.05 vs. control, and # P<0.05 vs. NC. We analyzed chemotaxis in NK-92 cells in response to varying concentrations of galectin-9 (G). A migration index (MI) was used to account for the high amount of random migration that occurred in the NK cells. IL-12 was used as a positive control for initiating chemotaxis in NK-92 cells. * P<0.05 vs. control, ** P<0.001 vs. control. Representative data are shown from at least 3 experiments.

The above results suggest an association between galectin-9 expression and NK cell infiltration in colon cancer tissue. The effects of galectin-9 on NK cells were next examined in vitro. We first explored whether the secretion of galectin-9 from tumor cells had a chemotactic effect on migration in NK cells. In these experiments, we determined the level of galectin-9 expression in 3 colon cancer cell lines, including SW480, SW620 and HT29, using qRT-PCR and western blot analysis. The results showed that HT29 cells had the highest galectin-9 expression level among these 3 cell lines (Fig 4A and 4B) and these cells were therefore selected for the following test. We designed siRNA to knock down galectin-9 expression in HT29 cells. As shown in Fig 4C–4E, galectin-9 mRNA, and protein levels and the concentration of galectin-9 in the culture supernatant were significantly decreased by two different siRNA sequences, siRNA #378 and siRNA #690. Interestingly, the HT-29 culture supernatants induced significantly enhanced chemotaxis in NK-92 cells, which are CD56^+^ human NK cells [19]. These effect of the HT-29 supernatants on NK-92 chemotaxis was significantly reversed by the addition of the galectin-9 siRNAs#378 and #690 groups (Fig 4F).Then by using another colon carcinoma cell line SW620, we consistently found that galectin-9 secreted from SW620 also had a chemotactic effect on NK-92 cells migration (S1 Fig).

Next, we examined whether galectin-9 is a chemoattractant for NK cells. In these experiments, different doses of rh-galectin-9 were analyzed to determine galectin-9’s chemotactic effect on NK-92 cells. The results showed that 25 and 50 ng/mL rh-galectin-9 induced chemotaxis in NK-92 cells but that the difference was not significant. At a concentration of 100 ng/mL, NK-92 cell migration was specifically increased across the transwell membrane by approximately 2-fold (P<0.001, compared to the control, Fig 4G).

### Galectin-9 promotes F-actin rearrangements in NK cells by enhancing Rho family expression in NK cells

**Fig 5:**
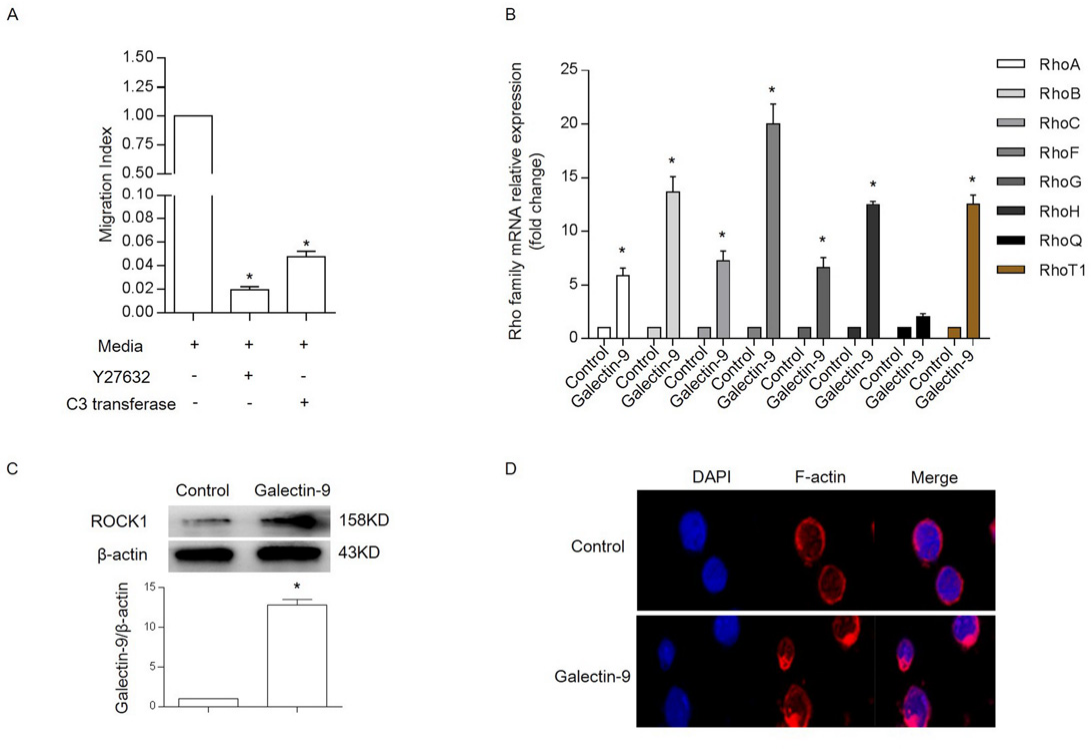
Galectin-9 promotes F-actin rearrangement by enhancing the expression of Rho family members and ROCK1. (A), Chemotaxis was inhibited by Rho and ROCK inhibitors in NK-92 cells. NK-92 cells were stimulated using medium alone for 4 h or medium containing Y27632 (20 μM) or C3 transferase (2 μg/mL) for 1.5 h. A migration index (MI) was used to account for the high random migration that was observed in NK cells. * P<0.05 vs. control. (B), The expression of RhoA, RhoB, RhoC, RhoF, RhoG, RhoH, RhoQ and RhoT1 mRNA using RT-PCR in NK-92 cells that were incubated in the presence or absence of rh-galectin-9 at a concentration of 100 ng/mL for 4 h; * P<0.05 vs. control. (C), The expression of ROCK1 protein in NK-92 cells that were incubated in the presence or absence of rh-galectin-9 at a concentration of 100 ng/mL for 24 h. (D), Representative micrographs of NK-92 cells that were incubated in the presence or absence of rh-galectin-9 at a concentration of 100 ng/mL for 4 h. F-actin was stained with rhodamine-labeled phalloidin (red), and cell nuclei were labeled with DAPI (blue) (630×). Representative data are shown from at least 3 experiments.

Lymphocytes undergo dramatic cytoskeletal rearrangements during cell migration.The small GTPase Rho is a pivotal regulator of the actin cytoskeleton, and Rho kinase, which is also known as Rho-associated coiled coil kinase (ROCK), is a Rho effector protein. To assess the involvement of Rho/ROCK in the transmigration of NK cells, chemotaxis assays were performed in the presence of the well-characterized ROCK inhibitor, Y27632, and another Rho-specific inhibitor, C3 transferase. The results showed that Y27632 and C3 transferase significantly inhibited NK-92 cell migration (Fig 5A).

The above results showed that galectin-9 regulates NK cell migration. Therefore, we next examined the effects of rh-galectin-9 on Rho family-related molecules in NK cells. The results showed that rh-galectin-9 significantly increased the expression of multiple members of the Rho family, including RhoA, RhoB, RhoC, RhoF, RhoG, RhoH, and RhoT1 (Fig 5B). As shown in Fig 5C, ROCK1 protein levels were significantly up-regulated in NK cells that were stimulated with rh-galectin-9 for 24 h.These results suggested that galectin-9 regulates a variety of Rho family molecules in NK cells.

We next examined whether galectin-9 influences rearrangements in the F-actin cytoskeleton in NK-92 cells. NK-92 cells were treated with rh-galectin-9 at a concentration of 100 ng/mL for 4 h, and changes in F-actin polymerization in NK-92 cells were subsequently examined. In the control group, the F-actin in NK-92 cells formed a distinct phalloidin-stained ring around the cell membrane. After treatment with rh-galectin-9, the F-actin became aggregated at one end of the cell, as shown in Fig 5D.

### Galectin-9 enhances primary NK cell chemotaxis

**Fig 6:**
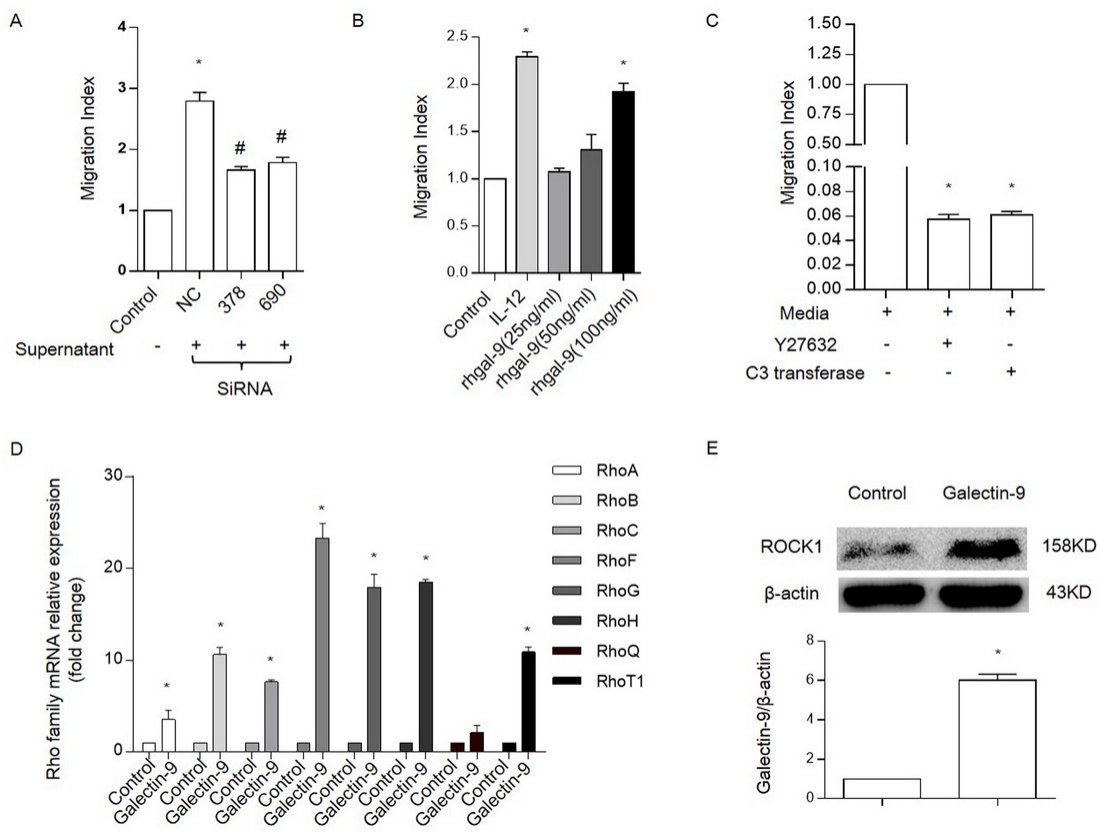
Galectin-9 enhances primary NK cell chemotaxis by increasing Rho family expression. Chemotaxis of primary NK cells in response to HT29 supernatants treated with galectin-9 siRNA(A) or varying concentrations of galectin-9(B). A migration index (MI) was used to account for the high amount of random migration that occurred in primary NK cells. IL-12 was used as a positive control for initiating chemotaxis primary NK cells. * P<0.05 vs. control. And # P<0.05 vs. NC. (C), Chemotaxis was inhibited by Rho and ROCK inhibitors primary NK cells. Primary NK cells were stimulated using medium alone for 4 h or medium containing Y27632 (20 μM) or C3 transferase (2 μg/mL) for 1.5 h. * P<0.05 vs. control. (D), The expression of RhoA, RhoB, RhoC, RhoF, RhoG, RhoH, RhoQ and RhoT1 mRNA using RT-PCR in primary NK cells that were incubated in the presence or absence of rhGalectin-9 at a concentration of 100 ng/mL for 4 h; * P<0.05 vs. control. (E), the expression of ROCK1 protein in primary NK cells that were incubated in the presence or absence of rhGalectin-9 at a concentration of 100 ng/mL for 24 h. Representative data are shown from at least 3 experiments.

In order to further validate our results, we perform the main experiments with primary NK cells. The results show that the migration of primary NK cells can be influenced by both galectin-9 secreted by HT29 (Fig 6A) or rh-galectin-9 (Fig 6B). Rho and ROCK inhibitors inhibit the chemotaxis of primary NK cell (Fig 6C). In addition, rh-galectin-9 also increase the expression of Rho family (Fig 6D) and ROCK1(Fig 6E).

## Discussion

In the present study, we demonstrated that galectin-9 expression is lower in colon tumor tissue than in corresponding normal tissues and galectin-9 levels are associated with NK cell infiltration and patient survival. The in vitro studies showed that galectin-9 increased NK cell recruitment by exerting effects on Rho/ROCK1 expression and F-actin polarization. We have therefore identified a new mechanism by which galectin-9 influences NK cell recruitment during colon tumor development, and this increases our current knowledge of the molecular involvement of galectin-9 in tumor immunity.

Galectin-9 has been observed to be down-regulated in various tumor types, such as melanoma[16], cervical squamous cell cancer[23], breast cancer[24]and hepatocellular carcinoma[25], and it potentially serves as a clinically significant biomarker for predicting outcomes in these patients. However, little is known about the details relating to the expression of galectin-9 in colon cancer tissues. Our results show that although the majority of colon cancer cells express galectin-9, both the positive rate and the level of expression are lower in these tissues than in normal colon tissues. In tumors, higher galectin-9 expression is associated with early TNM stage, no lymph node metastasis, good histological differentiation, and longer overall survival times. Importantly, these results suggest that this protein might be a promising biomarker for survival in patients with colon tumors.

The effects of galectin-9 in tumors are complex which are implicated in several aspects including tumor cell adhesion [15], apoptosis and cell cycle [16], migration [26], and angiogenesis [27]. Furthermore, it has been reported that galectin-9 exerted immunoregulatory roles in tumor microenvironment. Results showed that galectin-9 induced macrophages to differentiate into plasmacytoid dendritic cell-like macrophages[14, 28], which increased Tim-3^+^ dendritic cells and CD8^+^ T cells[29]. However, others studies demonstrated that galectin-9 played immunosuppressive roles by regulating immunosuppressive cells such as MDSC [30] and Tregs[31]. Lymphocyte infiltration into colorectal tumors has been associated with good prognoses[32, 33]. NK cells recognize and kill tumor cells, and they play an important role in tumor immune surveillance. Insufficient NK cell trafficking to tumor site might therefore be a novel mechanism for tumor escape[8, 34–36]. In the present study, we observed that significantly fewer CD56^+^ NK cells infiltrated colon tumor than adjacent normal tissues, and these results were consistent with the result of previous reports[10]. In addition, we also observed that the distribution of NK cells was correlated with galectin-9 expression. Previous studies using tumor-bearing mice showed that administrating galectin-9 increased the numbers of NK cells in the intraperitoneal exudate and enhanced the cytotoxic activity of NK cells[14]. Together with our findings in colon cancer tissues, these data suggest that galectin-9 might be involved in the recruitment of NK cells. The role of galectin-9 as a chemoattractant for NK cells was partially demonstrated by our in vitro transwell studies, which showed that both colon cancer cell-derived galectin-9 and rh-galectin-9 enhanced the migratory properties of NK cells, whether they are NK-92 or primary NK cells. Interestingly, these experimental conditions had no significant effect on the viability and proliferation of NK cells (data not shown). In addition,it is inconsistent regarding the role of galectin-9 in regulating NK cell activity [37–40]. These studies suggested that the effects of galectin-9may be dependent on the microenvironment. We found galectin-9 can recruit NK cells to colon tissues in this paper. However, the functions and activities of recruited NK cells have not yet been well described and will need to explore in the future.

It has been shown that galectin-9 regulated NK cells activity via Tim-3 signal pathway. Tim-3(+) NK cells/NK-92 cells significantly increased IFN-γ production in response to soluble rh-galectin-9[37]. Moreover, galectin-9 can trigger Tim-3 downregulation on NK cells and lead to enhanced NK cell activity in HIV infection[41]. And at the maternal-fetal interface, the galectin-9/Tim-3 pathway is also involved in the regulation of NK cell function[39, 42].These results suggest that galectin-9/Tim-3 is an important pathway involved in NK cells activities. However, whether this pathway is involved in the regulation of NK cell recruitment and activation in colon cancer still needs to be confirmed in the future.

Notably, studies have suggested that posttranscriptional splicing may affect galectin-9 protein function. So far, 6 splice variants of galectin-9 have been identified based on exclusion of exons 5, 6 and 10[43, 44]. Splice variants lacking exon 5 or/and 6 displayed altered length of the linker domain between the two carbohydrate recognition domains (CRDs), which influences the rotational freedom of both CRDs and increase gal-9 valency[45, 46].Furthermore, other studies have reported splice variants that lack exon 10, such as gal-9Δ10, gal-9Δ5/10 and gal-9Δ5/6/10do not appear to be secreted[47]. As with our findings, further research is needed to explore what types of variant are involved in NK cells recruiting in colon cancer.

The coordinated reconstitution of the actin cytoskeleton is required for cell migration. The actin cytoskeleton senses and integrates chemical and physical signals into force-generating structures to control motility. Rho GTPase family proteins are key regulatory molecules that link surface receptors to the organization of the actin cytoskeleton in all the eukaryotic cells [48, 49].Rho/ROCK signaling is necessary for many cytoskeleton-dependent processes, including the regulation of the cytoskeleton by the phosphorylation of downstream substrates, increases in actin filament stabilization and the generation of actin-myosin contractility[50].In the current study, we demonstrated that galectin-9 can modulate Rho GTPase signaling in NK-92 cells or primary NK cells by increasing the expression of Rho family members and ROCK1. We also found that the F-actin cytoskeleton in NK-92 cells that were treated with galectin-9 appeared to undergo rearrangement, and to show no F-actin ring formation. Instead,in these cells, F-actin was aggregated at one end of the cell. The results suggested that galectin-9 regulates the F-actin cytoskeleton in NK cells. Similarly, galectin-8, another tandem-repeat galectin similar to the galectin-9 protein, induces cytoskeleton rearrangement in Jurkat T cells[51].However, to our knowledge, this study is the first study to demonstrate that galectin-9 induces F-actin rearrangement in NK cells.Specific mechanism for this process needs to be further explored in a future study.

In conclusion, these data provided the first evidence that galectin-9 is down-regulated in colon tumor tissue and suggests a poor outcome. Furthermore, we found that galectin-9 has a novel function in immunological surveillance by regulating F-actin polarization in NK cells, and this regulation is associated with the activation of Rho/ROCK1 signaling.These results provide a foundation for the future exploration of a potential role for galectin-9 in the regulation of NK cell trafficking, potentially representing a new target for the immune treatment of colon cancer.

## Supporting Information

S1 Fig Galectin-9 secreted by SW620 enhances NK cell chemotaxis. (TIF)

S1 File Supplementary Materials and methods (DOC). (DOCX)

S2 File Supplementary Figure legend. (DOCX)
