# Supplementary figures and images for: Reduced Expression of Galectin-9 Contributes to a Poor Outcome in Colon Cancer by Inhibiting NK Cell Chemotaxis Partially through the Rho/ROCK1 Signaling Pathway
Source: PLoS One. 2016 Mar 30;11(3):e0152599. doi: 10.1371/journal.pone.0152599 (PMC4814049; doi:10.1371/journal.pone.0152599)

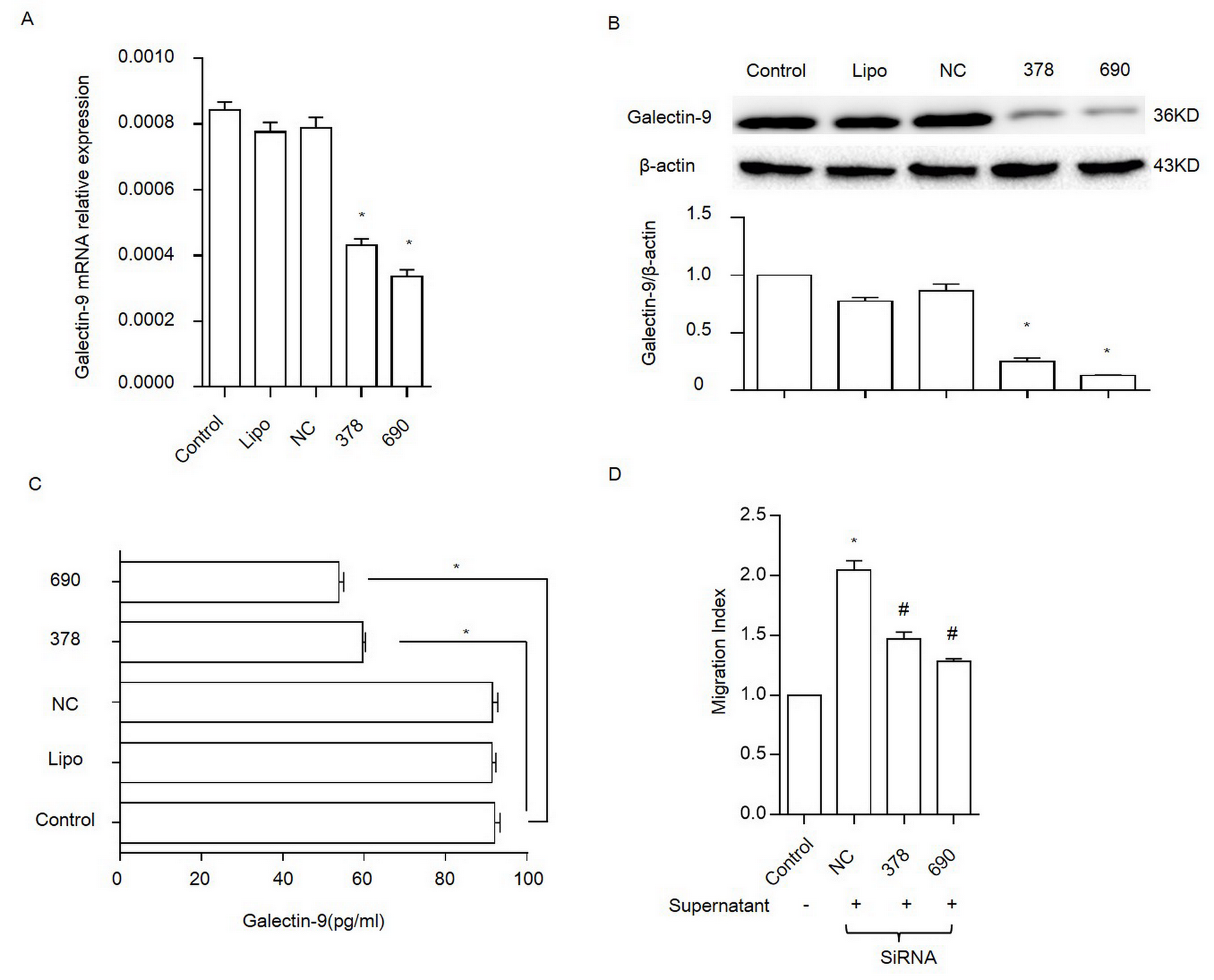

Supplement: S1 Fig — (TIF) [file pone.0152599.s001.tif]
