# Supplementary material for: Reduced Expression of Galectin-9 Contributes to a Poor Outcome in Colon Cancer by Inhibiting NK Cell Chemotaxis Partially through the Rho/ROCK1 Signaling Pathway
Source: PLoS One. 2016 Mar 30;11(3):e0152599. doi: 10.1371/journal.pone.0152599 (PMC4814049; doi:10.1371/journal.pone.0152599)
Supplement: S1 File — (DOCX) [file pone.0152599.s002.docx]

**Supplementary material**

**Patients**

A total of 128 colon tumor patients were enrolled in this study. Among these patients, 38 colon cancer and matched para-tumor tissue samples were obtained to evaluate differences in galectin-9 expression and NK cell distribution between the two sites. Basic information, including sex, age, and clinical data, was retrieved from the medicalrecords of each patient. An experienced pathologist reviewed the histopathologicalcriteria, including TNM stage (7th edition, Union for International Cancer Control). Information on survival (90 patients) was available from clinical reports. The key characteristics of the patients in the present study are summarized in Table 1. The follow up was completed in December 2014. The median survival time was 56 months (3-97 months). No patients received preoperative therapy.

**Evaluation of the results from immunohistochemical staining**

For galectin-9, the immunohistochemical score was determined based on the percentage of positive cells and staining intensity. The expression intensity was represented as a histological score (∑pi), where p is the percentage of galectin-9 positive cancer cells (scored as 1, 1–10%; 2, 11–50%; 3, 51–80%; or 4, 81–100%) and i represents the staining intensity (scored as 0, no staining; 1, weak staining; 2, moderate staining; or 3, strong staining). Strong (++++), moderate (+++), mild (++), weak (+) and negative (-) staining were 10-12, 7-9, 4-6, 1-3 and 0, respectively, and the score was reduced to a 2-tier system (0-1: low vs. 2/3/4: high) for statistical analysis according to the median.

For CD56, the degree of CD56^+^ NK distribution was determined for more than 10 high-power (200×) microscopic fields in each tissue sample. A total of 5 areas with the densest lymphocyte distribution were selected, and microphotographs were obtained. The numbers of CD56^+^ cells around the primary tumor cancer nest and para-tumor normal colon gland were counted. In each case, the mean index of CD56^+^ cells per single high-power field was counted, and this number was used for statistical analysis..

**Western blotting analysis**

A bicinchoninic acid protein assay kit (P0010S, [Beyotime Biotechnology](http://www.beyotime.com/), China) was used to test protein concentrations. Samples containing 20 µg of total protein were loaded into each lane of an 8% SDS-polyacrylamide gel for electrophoresis. The separated proteins were transferred to a polyvinylpyrrolidone membrane (Millipore, Bedford, MA, USA). The membranes were subsequently incubated with rabbit anti-galectin-9 polyclonal antibodies (1:500, SANTA), rabbit anti-Rock1 polyclonal antibodies (ab45171, 1:500, Abcam, Cambridge, Massachusetts, USA), and mouse anti-β-actin monoclonal antibodies (TA09, 1:1,000, ZSGB-BIO, Beijing, China) overnight at 4°C and probed with the appropriate secondary antibodies, respectively. Immune complexes were detected using the enhanced chemiluminescent (ECL) system (1316801, Millipore, Bedford, MA, USA) and imaged using Image Station 4000MM Pro (Carestream Health Inc. Canada). Protein bands were quantified by densitometry analysis using Image J software (National Institutes of Health, USA). The integrated density data of target protein were normalized against reference. The target/reference ratio represents the relative levels of each target protein.
