# Supplementary material for: Reduced Expression of Galectin-9 Contributes to a Poor Outcome in Colon Cancer by Inhibiting NK Cell Chemotaxis Partially through the Rho/ROCK1 Signaling Pathway
Source: PLoS One. 2016 Mar 30;11(3):e0152599. doi: 10.1371/journal.pone.0152599 (PMC4814049; doi:10.1371/journal.pone.0152599)
Supplement: S2 File — (DOCX) [file pone.0152599.s003.docx]

# Supplementary figure legend

**Galectin-9 secreted by SW620 enhances NK cell chemotaxis.** SW620 cells were divided into 5 groups: a control group (cultured with only medium), a lipo group (treated with Lipofectamine 2000), an NC group (transfected with the negative control siRNA), a galectin-9-siRNA (#378) group and a galectin-9-siRNA (#690) group. The knockdown efficacies of siRNAs targeting galectin-9 were examined using qRT-PCR (A) and western blot analysis (B). * P<0.05 vs. control. (C), Secreted levels of galectin-9 were measured using galectin-9 ELISA kits. All results are shown as the mean and SEM of quadruplicate experiments. (D) Chemotaxis of NK92 cells in response to SW620 supernatants treated with galectin-9 siRNA; * P<0.05 vs. control, and # P<0.05 vs. NC. Representative data are shown from at least 3 experiments.
